# An Audit of CT Chest Reports and Their Potential Impact on the Workup of Patients with Suspected Lung Cancer

**DOI:** 10.1155/2021/6647087

**Published:** 2021-06-05

**Authors:** Andrew Weinstock, Luke Jeagal, Chantal Savard, Jana Taylor, Anne V. Gonzalez

**Affiliations:** ^1^Respiratory Epidemiology and Clinical Research Unit, McGill University Health Centre Research Institute, Montreal, Quebec, Canada; ^2^Montreal Chest Institute, McGill University Health Centre, Montreal, Quebec, Canada; ^3^Department of Radiology, McGill University Health Centre, Montreal, Quebec, Canada

## Abstract

**Background:**

Quality gaps exist in the diagnostic evaluation of lung cancer patients. The initial CT chest guides the workup of patients with suspected lung cancer. We sought to determine how frequently CT reports provided guideline-concordant recommendations with regard to additional imaging studies and/or invasive diagnostic procedures*. Methods*. This was a retrospective study. The records of patients referred for investigation of suspected lung cancer between January 1, 2015, and June 30, 2016, were reviewed. Patients with confirmed lung cancer, for whom CT scan images and reports were available, are included. CT reports were reviewed, with attention to additional imaging studies and/or invasive diagnostic procedures suggested. These recommendations were examined against current guidelines for lung cancer diagnosis and staging, based on suspected disease stage.

**Results:**

One hundred forty-six patients are included in the analysis. Most patients were diagnosed with non-small-cell lung cancer (NSCLC), and 63% had advanced disease (stages III and IV). Only 12% of CT reports contained guideline-concordant recommendations for additional imaging studies, with PET scan suggested in only 6% of reports. Potential invasive diagnostic procedures were suggested in one fifth of CT reports, and only 58% of these recommendations were in keeping with current guidelines. In particular, transthoracic needle aspiration (TTNA) was suggested in 26% of patients despite advanced stage disease.

**Conclusion:**

Guideline-concordant recommendations for investigation of suspected lung cancer are rarely available on CT reports. This is true with respect to both imaging studies and invasive diagnostic procedures. Incorporation of more evidence-based suggestions may reduce quality gaps in lung cancer diagnosis and staging.

## 1. Introduction

Lung cancer is the leading cause of cancer death in Canadian men and women [1]. Disease stage portends prognosis and guides therapy. Complete staging requires both imaging studies (CT scan, PET scan ± brain imaging) and invasive diagnostic procedures to achieve the necessary tissue confirmation [2]. Invasive testing can often provide simultaneous confirmation of tissue diagnosis and disease stage; this approach is favored as it leads to a more efficient investigation process [3].

Despite the importance of accurate lung cancer staging, evidence suggests it is often poorly done [4, 5]. Recent studies, based on clinical and administrative databases, have highlighted significant quality gaps in the diagnosis and staging of lung cancer patients [6–8]. Suboptimal lung cancer staging has been associated with worse patient outcomes [9, 10].

CT scans of the chest play a key role in the initial evaluation of patients with suspected lung cancer. While less sensitive and specific than PET scanning for noninvasive assessment of the mediastinum, CT is more readily available. CT is frequently the only test available to guide physician decisions with regard to next diagnostic step(s) in the workup of a patient with suspected lung cancer [2]. We sought to determine how frequently CT reports provided guideline-concordant recommendations with regard to additional imaging studies and/or potential invasive diagnostic procedures in this setting.

## 2. Methods

This was a retrospective study. The Rapid Investigation Clinic (RIC) was established in 2010 to improve and accelerate the investigation of patients with suspected lung cancer [11], and a prospective database of all patients evaluated at the RIC has been maintained. An institutional registry of all patients with a pathological diagnosis of lung cancer was established in 2008. These two databases were cross-referenced to identify patients investigated at the RIC, and in whom a diagnosis of lung cancer was confirmed, between January 1^st^, 2015, and June 30^th^, 2016. The CT scan of the chest that triggered additional investigations for suspected lung cancer was considered the initial CT scan. The subgroup of patients in whom the initial CT scan of the chest was performed at the McGill University Health Centre (MUHC) constituted the study group.

Patient characteristics were extracted from electronic health records and included age, gender, lung cancer subtype, and disease stage. The date and type of all imaging studies and invasive diagnostic procedures performed were also recorded. Patients were staged according to the IASLC 7^th^ edition lung cancer staging system [12, 13], and the 3^rd^ edition American College of Chest Physicians (ACCP) guidelines for lung cancer diagnosis and staging were followed during the study period [2].

The reports and images of the CT scan of the chest that triggered further investigation for suspected lung cancer were reviewed in detail. The CT diagnostic findings were extracted, with associated clinical TNM stage. All suggestions by the reporting radiologist with regard to additional imaging studies, and possible invasive diagnostic procedures, were recorded. We then determined whether these recommendations appeared to be concordant with current guidelines for lung cancer diagnosis and staging, based on suspected disease stage.

The first invasive diagnostic procedure performed was then examined, according to clinical disease stage. For the subgroup of patients with TxN1-3M0 clinical stage, guidelines recommend that invasive mediastinal staging be performed as the first diagnostic procedure [2, 3]; guideline-concordant care was thus examined in this particular subgroup of patients.

The data was collected in Excel (Microsoft), and basic descriptive analyses were performed using SPSS (IBM). Continuous data is presented as mean ± SD, and proportions are presented as percentages. The MUHC Research Ethics Board approved the study (study number 2017–2775).

## 3. Results

**Figure 1:**
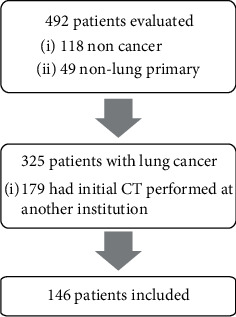
Flow diagram.

A total of 492 patients were evaluated at the RIC during the study period. Of these, 118 had a final nonmalignant diagnosis, and 49 cases were diagnosed with a non-lung primary malignancy. Amongst the remaining 325 patients, 179 had their initial CT scan performed outside the MUHC so that 146 patients constituted the study population (Figure 1). Patient baseline characteristics are detailed in Table 1. The mean patient age was 69.7 ± 8.8 years and 43% were women.

Recommendations for additional imaging studies and/or invasive testing embedded in CT reports are displayed in Table 2, respectively. The majority of CT reports (83%) contained no suggestions for additional imaging studies, while 12% of reports included guideline-concordant suggestions, such as PET/CT scanning (6%) or further tests to characterize suspected metastases. Notably, 5% of reports included guideline-discordant suggestions, in particular radiographic follow-up for nodules greater than 8 mm and/or associated with significant mediastinal lymphadenopathy. The majority of CT reports (79%) contained no recommendations with regard to invasive testing; 12% of reports had guideline-concordant suggestions, while 9% of reports had guideline-discordant suggestions. In particular, trans-thoracic needle aspiration (TTNA) was suggested in 26% of patients despite advanced stage disease.

The first invasive diagnostic procedures performed are reviewed in Table 3, according to clinical disease stage. Patients with Tx/N1-3/M0 constituted 40% of the study population. In this subgroup, 72% of patients underwent invasive mediastinal staging with a minimally invasive needle technique as their first diagnostic procedure.

## 4. Discussion

The initial CT scan of the chest has a pivotal role in guiding the subsequent evaluation of patients with suspected lung cancer. A CT scan is frequently the only imaging study available at initial evaluation; it provides key anatomical information with regards to the primary tumor and the extent of hilar and/or mediastinal adenopathy [2]. Physicians thus heavily rely on CT images and reports to determine disease stage and next diagnostic step(s). The current results suggest that CT reports only occasionally provide guidance in this regard.

The vast majority of CT reports failed to provide any recommendations, with only 12% of CT reports containing guideline-concordant suggestions of additional imaging studies and/or invasive diagnostic procedures. Also concerning is the finding of guideline-discordant suggestions in CT reports: a specific invasive procedure was suggested in only 1/5 reports, but, of these, a significant proportion were inappropriate.

The investigation of patients with lung cancer seeks to safely and efficiently establish disease stage, confirm tissue diagnosis, and ensure that sufficient tissue is acquired for molecular testing. The importance of accurate lung cancer staging is intuitive, as disease stage dictates treatment and informs prognosis [14]. The most recent ACCP guidelines emphasize the role of PET scanning in lung cancer staging. In addition, minimally invasive needle techniques (EBUS and EUS) have emerged as first-line invasive procedures for mediastinal staging [2]. An invasive approach that achieves concomitant tissue diagnosis and staging is recommended [2, 3].

There are limitations to this retrospective study. The study was based at a single university center, where a large number of patients with lung cancer are evaluated and treated annually, and dedicated chest radiologists reported the majority of CT scans of the chest. Yet, in this tertiary-care context, guideline-concordant recommendations were found in only 12% of the CT scan reports reviewed. It is unlikely that the community-based general radiologists would be more likely to provide guideline-concordant suggestions with regard to next steps in the workup of suspected lung cancer. Patients were staged according to the 7^th^ edition of the IASLC staging system, which was in use during the study period. The 8^th^ edition of the NSCLC staging system [15] entered North American clinical practice in 2018, but the nodal “N” classification has remained the same as in the 7^th^ edition, and the 3^rd^ edition ACCP staging guidelines remained in clinical use.

The downstream workup was reviewed in the subgroup of patients with hilar or mediastinal adenopathy: most patients underwent guideline-concordant invasive testing. However, these patients were evaluated in a specialized lung cancer investigation clinic, which likely limits the impact of a lack of recommendations in CT reports. When a patient develops symptoms that warrant further imaging, or suspicious findings are incidentally detected on CT, they generally present to their general practitioner or to community-based physicians. These physicians may be more likely to rely on CT chest reports to guide their decision-making with regard to further investigation and/or referral to the most appropriate specialist.

It could be argued that it is not the radiologist's place to provide recommendations with regard to downstream testing in a patient whose scan is suggestive of lung cancer. However, such recommendations are commonly included in the reports of CT scans performed for other indications. The Fleischner guidelines provide guidance as to the frequency of repeat imaging (and/or further management), based on size and density of incidentally detected lung nodule(s), and whether the patient is considered at low or high risk of malignancy [16]. The Lung-RADS categories similarly provide guidance with regard to management of findings detected on low-dose CT chest performed for lung cancer screening [17]. Radiologists could recommend evaluation of patients with suspected lung cancer in a specialized, rapid access clinic or consider direct referrals to such a program [18]. Specialized investigation clinics have been shown to improve timeliness and guideline-concordant care [11, 19].

“When you have a hammer, everything is a nail,” and radiologists may propose that a lung nodule is amenable to TTNA despite evidence of advanced disease. In fact, TTNA was suggested in 26% of patients whose CT report contained a specific recommendation with regard to an invasive diagnostic procedure, despite the presence of hilar and/or mediastinal adenopathy or distant metastases. TTNA has a high diagnostic yield for peripheral pulmonary lesions but is also associated with significant complications, including a 15–25% pneumothorax rate and 1–5% risk of hemorrhage [20–22]. In contrast, sampling of hilar or mediastinal nodes with EBUS carries a pneumothorax risk of 0.5% [23]. Proceeding first with TTNA in the presence of suspicious hilar and/or mediastinal nodes may expose the patient to unnecessary risks, particularly when invasive confirmation of nodal status will still be required.

Ost and colleagues reviewed the impact of diagnostic test sequencing on outcomes, using a retrospective cohort of over 15,000 patients with lung cancer. Only 21% of patients in whom mediastinal lymph nodes sampling would have been recommended as the first invasive test received guideline-consistent diagnostic evaluations. Yet, guideline-consistent care was found to be associated with significantly fewer procedures and complications, including pneumothoraces, chest tubes, hemorrhages and respiratory failure [10].

In conclusion, guideline-concordant recommendations for investigation of suspected lung cancer are rarely available on CT reports. This is true with respect to both imaging studies and invasive diagnostic procedures. The incorporation of more evidence-based suggestions in CT scan reports may contribute to reducing quality gaps in lung cancer diagnosis and staging, and this warrants further investigation.

## Figures and Tables

**Table 1:** Baseline patient characteristics.

Patient characteristics	Total *N* = 146 patients
Age (mean in years, ±SD)	69.7 (8.8)
Male sex, *N* (%)	85 (58)
NSCLC stage, *N* (%)	
I	32 (23)
II	20 (14)
III	40 (27)
IV	47 (32)
SCLC stage, *N* (%)	
Limited	2 (1)
Extensive	5 (3)

**Table 2:** Additional imaging studies and invasive testing suggested in CT reports.

	Total *N* = 146
*Additional imaging studies suggested in CT reports*	
No recommendation, *N* (%)	122 (83)
Guideline-concordant suggestion, *N* (%)	17 (12)
PET/CT	9
Abdominal imaging (US/MRI/CT)	5
Bone scan	2
Abdominal imaging and bone scan	1
Guideline-discordant suggestion, *N* (%)	7 (5)
Follow-up CT nodule >8 mm ± lymphadenopathy	7

*Invasive testing suggested in CT reports*	
No recommendation, *N* (%)	115 (79)
Guideline concordant testing, *N* (%)	18 (12)
TTNA: nodule (Tx/N0/M0)	7
TTNA: mediastinal mass (Tx/N1-3/M0)	1
Bronchoscopy (Tx/Nx/M1)*∗*	4
Tissue sampling, not specified (Tx/N0/M0) *∗*	6
Guideline discordant testing, *N* (%)	13 (9)
TTNA. nodule (Tx/N1-3/M0)	4
TTNA, nodule (Tx/Nx/M1)	4
Bronchoscopy (Tx/Nx/M1)*∗∗*	2
Tissue sampling, not specified (Tx/N1-3/M0) *∗∗*	2
Tissue sampling, not specified (Tx/Nx/M1)*∗∗*	1

*∗*Bronchoscopy was considered guideline-concordant in the presence of widely metastatic disease and endobronchial disease. Similarly, tissue sampling, not specified, was considered concordant in the presence of only one potential biopsy site. *∗∗*Bronchoscopy was considered guideline-discordant in the presence of an otherwise easily accessible biopsy site (e.g., nodes accessible with endoscopic ultrasound or accessible metastatic site) and no clear endobronchial disease. Similarly, tissue sampling, not specified, was considered guideline-discordant in the absence of a clear recommendation to target a specific, accessible biopsy site.

**Table 3:** First invasive test performed.

	Patient population (*N* = 146)
Tx/N0/M0*∗*, *N* (%)	38 (26)
TTNA	13
Bronchoscopy	7
EBUS	8
Surgery	10
Tx/N1-3/M0*∗*, *N* (%)	58 (40)
EBUS	39
Bronchoscopy with TBNA	2
TTNA, mediastinal mass	1
TTNA, nodule/mass	5
Bronchoscopy without TBNA	11
Tx/Nx/M1*∗*, *N* (%)	50 (34)
Thoracentesis	5
TTNA	7
Bronchoscopy	13
EBUS	20
EUS	2
Other (bone, extrathoracic lymph node, or distant site)	3

*∗*Clinical stage based on CT ± PET/CT results.

## Data Availability

Denominalized data are available from the authors upon reasonable request.

## Abbreviations

ACCP: American College of Chest Physicians
CT: Computerized tomography
EUS: Endoscopic ultrasound
EBUS: Endobronchial ultrasound
IASLC: International Association for the Study of Lung Cancer
PET: Positron emission tomography
RIC: Rapid investigation clinic
TTNA: Transthoracic needle aspiration.

## References

[1] Canadian Cancer Statistics Advisory Committee (2019). *Canadian Cancer Statistics: 2019*.

[2] Silvestri G. A., Gonzalez A. V., Jantz M. A. (2013). Methods for staging non-small cell lung cancer. *Chest*.

[3] Ost D. E., Jim Yeung S.-C., Tanoue L. T., Gould M. K. (2013). Clinical and organizational factors in the initial evaluation of patients with lung cancer. *Chest*.

[4] Detterbeck F. (2009). What is quality and does it matter?. *Journal of Thoracic Oncology*.

[5] Detterbeck F. C. (2012). The fable of Babel and building a foundation for quality. *Journal of Thoracic Oncology*.

[6] Almeida F. A., Casal R. F., Jimenez C. A. (2013). Quality gaps and comparative effectiveness in lung cancer staging. *Chest*.

[7] Ost D. E., Niu J., Elting L. S., Buchholz T. A., Giordano S. H. (2014). Determinants of practice patterns and quality gaps in lung cancer staging and diagnosis. *Chest*.

[8] Flanagan M. R., Varghese T. K., Backhus L. M. (2015). Gaps in guideline-concordant use of diagnostic tests among lung cancer patients. *The Annals of Thoracic Surgery*.

[9] Farjah F., Flum D. R., Ramsey S. D., Heagerty P. J., Symons R. G., Wood D. E. (2009). Multi-modality mediastinal staging for lung cancer among medicare beneficiaries. *Journal of Thoracic Oncology*.

[10] Ost D. E., Niu J., Elting L. S., Buchholz T. A., Giordano S. H. (2014). Quality gaps and comparative effectiveness in lung cancer staging and diagnosis. *Chest*.

[11] Ezer N., Navasakulpong A., Schwartzman K. (2017). Impact of rapid investigation clinic on timeliness of lung cancer diagnosis and treatment. *BMC Pulmonary Medicine*.

[12] Goldstraw P., Crowley J., Chansky K. (2007). The IASLC lung cancer staging project: proposals for the revision of the TNM stage groupings in the forthcoming (seventh) edition of the TNM Classification of malignant tumours. *Journal of Thoracic Oncology*.

[13] Goldstraw P. (2009). *International Association for the Study of Lung Cancer (IASLC) Staging Manual in Thoracic Oncology*.

[14] Detterbeck F. C., Boffa D. J., Kim A. W., Tanoue L. T. (2017). The eighth edition lung cancer stage classification. *Chest*.

[15] Goldstraw P., Chansky K., Crowley J. (2016). The IASLC lung cancer staging project: proposals for revision of the TNM stage groupings in the forthcoming (eighth) edition of the TNM classification for lung cancer. *Journal of Thoracic Oncology: Official Publication of the International Association for the Study of Lung Cancer*.

[16] MacMahon H., Naidich D. P., Goo J. M. (2017). Guidelines for management of incidental pulmonary nodules detected on CT images: from the fleischner society 2017. *Radiology*.

[17] Radiology ACo. Lung CT Screening Reporting & Data System (Lung-RADS) Version 1.1, https://www.acr.org/Clinical-Resources/Reporting-and-Data-Systems/Lung-Rads

[18] Veenstra J. S., Khalid T., Stewart K. C. (2020). Automatic referral for potential thoracic malignant diseases detected on computed tomographic scan. *The Annals of Thoracic Surgery*.

[19] Laerum D., Brustugun O. T., Gallefoss F., Falk R., Strand T.-E., Fjellbirkeland L. (2020). Reduced delays in diagnostic pathways for non-small cell lung cancer after local and national initiatives. *Cancer Treatment and Research Communications*.

[20] Heerink W. J., de Bock G. H., de Jonge G. J., Groen H. J. M., Vliegenthart R., Oudkerk M. (2017). Complication rates of CT-guided transthoracic lung biopsy: meta-analysis. *European Radiology*.

[21] Sharma A., Shepard J.-A. O. (2018). Lung cancer biopsies. *Radiologic Clinics of North America*.

[22] Wiener R. S., Schwartz L. M., Woloshin S., Welch H. G. (2011). Population-based risk for complications after transthoracic needle lung biopsy of a pulmonary nodule: an analysis of discharge records. *Annals of Internal Medicine*.

[23] Eapen G. A., Shah A. M., Lei X. (2013). Complications, consequences, and practice patterns of endobronchial ultrasound-guided transbronchial needle aspiration. *Chest*.

